# Low temperature vaporized hydrogen peroxide sterilization of 3D printed devices

**DOI:** 10.1186/s41205-024-00206-1

**Published:** 2024-02-28

**Authors:** Randal Eveland, Kathleen Antloga, Ashley Meyer, Lori Tuscano

**Affiliations:** STERIS, 5960 Heisley Road, Mentor, OH 44060 USA

**Keywords:** 3D printing, Sterilization, Vaporized hydrogen peroxide

## Abstract

**Background:**

Low temperature vaporized hydrogen peroxide sterilization (VH2O2) is used in hospitals today to sterilize reusable medical devices. VH2O2 sterilized 3D printed materials were evaluated for sterilization, biocompatibility and material compatibility.

**Materials & methods:**

Test articles were printed at Formlabs with BioMed Clear™ and BioMed Amber™, and at Stratasys with MED610™, MED615™ and MED620™. Sterilization, biocompatibility and material compatibility studies with 3D printed materials were conducted after VH2O2 sterilization in V-PRO™ Sterilizers. The overkill method was used to evaluate sterilization in a ½ cycle. Biocompatibility testing evaluated the processed materials as limited contact (< 24-hours) surface or externally communicating devices. Material compatibility after VH2O2 sterilization (material strength and dimensionality) was evaluated via ASTM methods and dimensional analysis.

**Results:**

3D printed devices, within a specific design window, were sterile after VH2O2 ½ cycles. After multiple cycle exposure, the materials were not cytotoxic, not sensitizing, not an irritant, not a systemic toxin, not pyrogenic and were hemo-compatible. Material compatibility via ASTM testing and dimensionality evaluations did not indicate any significant changes to the 3D printed materials after VH2O2 sterilization.

**Conclusion:**

Low temperature vaporized hydrogen peroxide sterilization is demonstrated as a suitable method to sterilize 3D printed devices. The results are a subset of the data used in a regulatory submission with the US FDA to support claims for sterilization of 3D printed devices with specified materials, printers, and device design ^1^.

## Background

The hospital production of 3D printed, or additive manufacturing (AM), devices is becoming more prevalent. Patient specific 3D printed anatomical models are of tremendous value to medical practitioners for patient education, pre-operative surgical planning, surgeon training as well as interoperative and surgical use [2, 3]. With the increase in the number of evidence-based use cases, there are more clinical scenarios where anatomic models are beneficial in the procedure or operating room and this use requires sterilization. Patient specific 3D printed surgical guides improve patient outcomes via shorter procedure times and better post-operative results, and they are a prerequisite for many procedures. Sterilization prevents contamination of an established sterile field, and it mitigates patient risk when the medical team uses the surgical guide intraoperatively or interacts with the model during a procedure.

When a hospital produces a 3D printed medical device for patient use, [4, 5] the provider assumes the role as the medical device’s manufacturer, including the design, fabrication, sterilization, and surgical use. The process to create patient specific medical devices from patient radiological data has been well discussed [3, 6]. One area of this process that has been less studied and documented is the impact of sterilization on devices and the resulting data to support a general approach to sterilization within a healthcare setting.

For sterilization, steam is the most common method identified for hospital manufactured 3D printed devices. Sterilization using low temperature vaporized hydrogen peroxide (VH2O2) has recognized benefits for temperature and moisture sensitive medical devices. The operating temperature for vaporized hydrogen peroxide sterilizers is typically about 50 °C while typical steam sterilization exposure temperatures can range from 121 to 134 °C and devices can reach these temperatures during the sterilization cycle.

The purpose of this study is to report test data for microbicidal efficacy, material compatibility, and biocompatibility testing performed in the low temperature vaporized hydrogen peroxide VPRO maX 2 Sterilizer Specialty Cycle.

## Methods

### Test articles

Test-specific 3D printed samples used as test articles were with vat polymerization (Form3B, Formlabs, Cambridge, MA) using BioMed Clear and BioMed Amber resins and with material jetting (J750, Stratasys, Israel) with MED610, MED615, and MED620 resins. The test articles were printed, cured, cleaned, and support material removed in accordance with printer instructions for use (contact printer manufacturers or see their websites at www.formlabs.com and www.stratasys.com).

The Formlabs vat polymerization printers employ a laser beam to cure liquid resin into hardened plastic via photopolymerization. The vat polymerization creates bonds within and between print layers, and in conjuncture with the final curing process, creates an anisotropic part that should not contain voids. The Stratasys material jetting printers layer photo-polymeric materials from printer heads where they are subsequently cured on exposure to UV light creating isotropic materials without voids.

Microbicidal efficacy and biocompatibility evaluations used a proprietary test article (3DTA or 3-Dimensionally Printed Test Article) that contained worst-case features of surgical guides and anatomical models to allow evaluation in accordance with the sterilization cycle design limits. The design included a lumen (tubular, hollow) feature that established the 3D printed medical device design limitation within efficacy evaluations (3 mm ID x 20 mm length or 3 mm ID x 30 mm length, dependent on material) and a variety of surface features present on surgical guides and anatomical models. For ASTM material compatibility evaluations, a 3D printed test article was printed to meet each method’s specific test article requirement.

### Sample processing

3D printed test samples were exposed to either a V-PRO maX 2 Sterilizer Specialty Cycle, a V-PRO maX 2 Specialty ½ Cycle, or to a worst-case chemical exposure of three [3]. VPRO s2 Sterilizer Lumen Cycles. VAPROX HC Sterilant was used for all evaluations. Specialty Cycle selection (D, E, or F) is based on the material used as each material is qualified for use in a specific cycle as shown in Table 1.

The worst-case chemical exposure condition is determined in terms of hydrogen peroxide (H_2_O_2_) theoretical sterilant dose (mg-min/L). The dose is calculated by multiplying the theoretical concentration (in mg/L; 9.1 mg/L H_2_O_2_ for the V-PRO maX 2 Sterilizer and 10.8 mg/L H_2_O_2_ for V-PRO s2 Sterilizer) by the sterilant exposure time (min). The sterilant exposure phase of each Specialty Cycle is the same, with two [2] sterilization pulses and a total sterilant exposure time of 7.5 min for a theoretical hydrogen peroxide sterilant dose of 68 mg/L x min (7.5 min x 9.1 mg/L = 68 mg/L x min). The V-PRO s2 Sterilizer Lumen Cycle has the highest theoretical sterilant dose (346 mg-min/L; 32 min x 10.8 mg/L) of the V-PRO Sterilizers’ cycles [7]. For the worst-case chemical exposure, unpackaged 3D printed test samples were placed in a tray bottom without a lid or any additional load, and exposed to three back-to-back V-PRO s2 Sterilizer Lumen Cycles for a total dose of 1038 mg/L x min (3 × 346 mg-min/L). The 1038 mg/L x min dose equates to a 15-fold higher chemical exposure than the Specialty Cycle.

### Sterilization efficacy

When designing experiments to demonstrate device sterility following manufacturing, one method considers the known bioburden of the manufacturing process while another method uses an overkill approach (see ISO 22441:2022 *Sterilization of health care products — Low temperature vaporized hydrogen peroxide — Requirements for the development, validation and routine control of a sterilization process for medical devices* Annex B and D). For this study, the overkill method was used. The overkill method is common for devices to be processed in hospitals and for sterilization of single-use devices in industry. The method used ≥ 10^6^ of the most resistant organism to the low temperature vaporized hydrogen peroxide process, *Geobacillus stearothermophilus*, in a ½ cycle under worst-case processing conditions. Verification in a ½ cycle, with half the sterilization exposure time, validates the full-cycle sterilization process with a 10^6^ sterility assurance factor.

The 3DTA was used to evaluate for surface and lumen sterilization in triplicate trials. Each test site was challenged with ≥ 1 × 10^6^ colony forming units (CFU) *Geobacillus stearothermophilus* spores and dried. The test articles were pouched along with a sterilization load and exposed to a Specialty ½ Cycle. The ½ cycle uses the same Condition and Aeration Phase as the standard cycle, but ½ the sterilant exposure. After sterilization, the test articles were aseptically cultured into tryptic soy broth, incubated for 14 days at 55–60 °C, then evaluated for growth.

### Biocompatibility

3DTAs of each material were pouched, placed in a tray bottom without a lid or any additional load and exposed to three back-to back Specialty Cycles. Following processing, the 3DTA were extracted with a mixed polarity solvent (cell culture test sample media) in accordance with ISO 10993-5 and ISO 10993-12. A cytotoxicity evaluation in accordance with ISO 10993-5 was conducted.

For the remaining biological evaluations, material coupons were pouched, placed in a tray bottom without a lid or any additional load and exposed to three Specialty Cycles, then evaluated by NAMSA test laboratories for sensitization (ISO 10993-10), intracutaneous irritation testing (ISO 10993-23), systemic toxicity (ISO 10993-11), material mediated pyrogenicity (ISO 10993-11) and hemocompatibility (ISO 10993-4 and ASTM F756).

### Chemical evaluations

For the chemical analysis of 3D printed materials post-processing, test articles were processed via a worst-case chemical exposure (see Sample Processing section). Exposed and unexposed samples were then analyzed by Fourier Transform Infrared Spectroscopy (FTIR), Gas Chromatography/ Mass Spectroscopy (GC-MS), and Inductively Coupled Plasma (ICP) atomic emission spectroscopy. The FTIR evaluated sample surfaces while the GC-MS and ICP evaluated 24-hour (37 °C) test article water extracts in accordance with ISO 10993-12 extraction recommendations. GC-MS was conducted on the chloroform soluble components of the water extract.

### Sterilant Residue

The residual hydrogen peroxide sterilant remaining post sterilization was evaluated after exposure of the pouched 3DTA without any additional load to three Specialty Cycles. The test articles were extracted at 37 °C in sterile water for 24 to 72 h and analyzed for hydrogen peroxide residue by a validated (in accordance with USP < 1225>) xylenol orange spectrophotometric assay. The basis of the assay is the complexing of ferric ion (Fe 2+) by H_2_0_2_ in the presence of xylenol orange (CAS Number 3618-43-7). Peroxides in the sample oxidize Fe 2 + to Fe 3+, and the Fe 3 + forms a colored complex with xylenol orange that is read at 525 nm.

### Material evaluations

ASTM test-specific 3D printed test articles for tensile strength, flexural strength, compressive strength, Izod notched impact, and Shore hardness were processed via a worst-case chemical exposure (see Sample Processing section). The number of test articles processed and tested was in accordance with ASTM test-specific requirements. Additionally, a single Specialty Cycle exposure was used to evaluate a subset of test articles in a simulated use exposure. For all material evaluation exposures, unpackaged test articles (to allow for maximum exposure to sterilant) were placed in a tray base without any additional load. Post-exposure, the exposed and unexposed test articles were sent to Westmoreland Mechanical Testing & Research, Inc. for evaluation.

ASTM test results were evaluated via ANOVA, General Linear Model analysis and by Tukey Pairwise Comparison for statistical significance (*p* < 0.05). Statistical analyses were conducted with Minitab 19.2020.

Pre- and post-sterilization dimensional analysis was conducted with a 3DTA printed with each Formlabs and Stratasys material. Each 3DTA was exposed to the Specialty Cycle identified for the material. Dimensional analyses were conducted via physical measurements (calipers) and via scanning with a Faro inspection arm/digital scanner before and after sterilization. Twenty to twenty-seven physical measurements were made for each 3DTA. Scanner data was processed with PolyWorks™ 2019 Inspector Essentials software and evaluated for differences.

The temperature of plastic medical devices was evaluated before and after sterilization to determine the impact of the longest cycle, Specialty F Cycle, on temperature. An infrared thermometer was used to determine temperature before and after sterilization in three independent evaluations.

## Results

### Sterilization efficacy

3DTAs of each material were evaluated over triplicate trials for surface and lumen sterilization efficacy. In each trial, two lumen sites and six total combined surface features were tested. All test articles were sterile after exposure to Specialty ½ Cycles (Table 1). These results demonstrate that the Specialty Cycle effectively sterilizes 3D printed surgical guides and anatomical models made with the tested Formlabs and Stratasys materials within the design limitations evaluated. The lumen results qualify devices with equivalent or larger ID and equivalent or shorter length, e.g., ≥ 3 mm ID x ≤ 30 mm length for Formlabs BioMed Amber.

**Table 1:** Specialty ½ cycle microbicidal efficacy evaluation results

Material	Specialty Cycle	Lumens		Surfaces
		Lumen Dimension	# Sterile/# Tested	# Sterile/# Tested
Formlabs BioMed Amber	F	3 mm ID x 30 mm length	6/6	18/18
Formlabs BioMed Clear	D	3 mm ID x 30 mm length	6/6	18/18
Stratasys MED610	E	3 mm ID x 20 mm length	6/6	18/18
Stratasys MED615	E	3 mm ID x 20 mm length	6/6	18/18
Stratasys MED620	E	3 mm ID x 20 mm length	6/6	18/18

### Biocompatibility

The Formlabs BioMed Clear and BioMed Amber and the Stratasys MED610, MED615, and MED620 materials used in this study are identified as biocompatible by Formlabs and Stratasys. For sterilization in low temperature vaporized hydrogen peroxide, test devices and materials were processed in a sterilization cycle designed to ensure biocompatibility. Sterilization cycles from approximately 1 to 20 h were evaluated to determine the aeration required for the 3D printed devices to be safe for immediate use after sterilization. Once the aeration for each material was determined, biological evaluations were conducted. The Sterilize Phase was kept constant to ensure the same exposure of hydrogen peroxide sterilant (7.5 min). The importance of hydrogen peroxide sterilant removal through aeration is known in sterilization and in other vaporized hydrogen peroxide applications such as room decontamination [8]. It was apparent that 3D printed devices from these materials required more aeration than reusable medical devices processed in hospitals, which is typically 3 or 6 min aeration for similar sterilant exposure [7]. The Specialty D, E, and F Cycles identified for each material (Table 1) are approximately 8, 16, and 20 h, respectively, with the bulk of the time in the aeration phase (approximately 16 min of each cycle are used for conditioning and sterilization).

In accordance with ISO 10993-1, 3D printed surgical guides and anatomical models were categorized for use as surface and external communicating medical devices with limited duration (< 24 h) patient contact via mucosal membrane, breached or compromised surface, blood path (indirect), circulating blood, or tissue/bone/dentin.

Biological evaluations were conducted in accordance with the ISO 10,993 standard series for the biological evaluation of medical devices and were selected via a risk-based approach. As shown in Table 2, this included evaluation of Cytotoxicity, Sensitization, Intracutaneous Irritation, Systemic Toxicity, Material Mediated Pyrogenicity, and Hemocompatibility. The evaluations included both *in-vitro* (cell) and *in-vivo* (animal) testing.

All test controls responded as expected in these evaluations and there were no anomalous observations. The results in Table 2 show that the materials are not a biological safety concern after sterilization in the Specialty Cycle identified for each material.

**Table 2:** Biocompatibility results after 3x specialty cycle exposure

Material	Evaluation					
	Cytotoxicity* ISO 10993-5	Sensitization** ISO 10993-10	Intracutaneous Irritation testing** ISO 10993-23	Systemic Toxicity** ISO 10993-11	Material Mediated Pyrogenicity** ISO 10993-11	Hemocompatibility** ISO 10993-4 and ASTM F756
Formlabs BioMed Amber	Not cytotoxic	Not sensitizing	Not an irritant	Not a systemic toxin	Not pyrogenic	Hemo-compatible
Formlabs BioMed Clear	Not cytotoxic	Not sensitizing	Not an irritant	Not a systemic toxin	Not pyrogenic	Hemo-compatible
Stratasys MED610	Not cytotoxic	Not sensitizing	Not an irritant	Not a systemic toxin	Not pyrogenic	Hemo-compatible
Stratasys MED615	Not cytotoxic	Not sensitizing	Not an irritant	Not a systemic toxin	Not pyrogenic	Hemo-compatible
Stratasys MED620	Not cytotoxic	Not sensitizing	Not an irritant	Not a systemic toxin	Not pyrogenic	Hemo-compatible

* Testing conducted at STERIS in accordance with ISO 10993-5 standard under Good Laboratory Practice (GLP) regulations as provided in 21 CFR § 58

** Testing conducted at NAMSA in accordance with the identified ISO 10,993 standards. NAMSA is certified to ISO 9001:2015 and is accredited to ISO/IEC 17025:2017

### Chemical evaluations

Chemical evaluations were completed to understand potential differences in materials after vaporized hydrogen peroxide sterilization. The testing included an extensive chemical analysis of materials pre- and post-sterilization. As a worst-case, the materials were exposed to vaporized hydrogen peroxide at 15-times higher concentration than that used for sterilization (see Sample Processing section). The intent of these evaluations was not to characterize the material, but instead to look for any differences caused by the extreme worst-case exposure.

A detailed evaluation of FTIR surface spectrum of the materials did not identify any differences caused by the chemical exposure. In the GC-MS and ICP analyses, exposed and unexposed materials were extracted for analysis. Analysis of the GC-MS and ICP results similarly did not identify that any new materials were created from reaction with the hydrogen peroxide sterilant.

### Sterilant residue

To further evaluate the safety of vaporized hydrogen peroxide exposed Formlabs and Stratasys materials, test devices processed for three sterilization cycles (as identified in Table 1) were extracted and tested for hydrogen peroxide residuals. Table 3 shows the residual hydrogen peroxide was less than 0.3 mg hydrogen peroxide per gram per device. These material residual levels were determined to be less than the tolerable exposure limits for mucosal and internal tissue contact established according to ISO 10993-17. As a relative comparison, a 3% hydrogen peroxide solution (30,000 ppm H_2_O_2_ or 30 mg H_2_O_2_ per gram of water) is identified as a topical solution per USP (US Pharmacopeia).

**Table 3:** Hydrogen peroxide sterilant residue after 3x specialty cycle exposure and 72-hour extraction

Material	Specialty Cycle	mg H_2_O_2_/g Device
Formlabs BioMed Amber	F	0.27
Formlabs BioMed Clear	D	0.22
Stratasys MED610	E	0.13
Stratasys MED615	E	0.12
Stratasys MED620	E	0.12

These evaluations support that test articles produced with Formlabs BioMed Clear and BioMed Amber and Stratasys with MED610, MED615 and MED620 retain biocompatibility after vaporized hydrogen peroxide sterilization.

### Material evaluations

#### ASTM testing

Materials evaluations in this study were conducted in consideration of ISO/ASTM 52910:2018(E) *Additive manufacturing — Design — Requirements, guidelines and recommendations*. Shore Hardness, a common material test, was also evaluated. For each ASTM test evaluated, a test article was 3D printed to meet the method’s test article requirement. To understand the potentially differing impacts of worst-case hydrogen peroxide sterilant exposure versus the sterilization process (e.g. pressure, temperature, and sterilant) on the material, evaluations were conducted with a worst-case chemical exposure to hydrogen peroxide and, for select tests, with a simulated use exposure after processing in the Specialty Cycle identified for each material (Table 1).

Exposed test articles and unexposed control test articles were evaluated in accordance with ASTM methods at an external contract laboratory. There were no unusual observations or non-conformities identified in any of the contract laboratory test reports. Test results were analyzed for statistical differences using Minitab statistical analysis software. When test articles were required to be printed in two test configurations, e.g., in both lengthwise and crosswise print configurations per the ASTM test method, an ‘H’ indicates the sample was printed in a horizontal/lengthwise configuration (the XY plane) and a ‘V’ indicates the sample was printed 90° to this configuration in the vertical/crosswise configuration (the XZ plane).

Tables 4 and 5 show ASTM test results as a percent difference comparison of exposed test articles to the unexposed control test articles for the worst-case chemical exposure and simulated use exposure respectively. Percent change was calculated as follows: (exposed – unexposed)/unexposed x 100%. As identified in Tables 4 and 5 by an asterisk, many exposed test article results were not statistically different from the unexposed control test article. Increases in strength after processing are not considered practically significant. The remaining results, where material changes were negative post-processing, are not considered practically significant as the overall data does not suggest any gross negative material effects. Practical significance could vary depending on the specific device design, so the device manufacturer must ensure the printed device design will meet its intended use.

**Table 4:** Mechanical property evaluations after worst-case exposure to vaporized hydrogen peroxide

Test Name	ASTM^†^	Material / % change				
		Formlabs BioMed Amber	Formlabs BioMed Clear	Stratasys MED610	Stratasys MED615	Stratasys MED620
Tensile Strength	D638	-8.5% (V) -3.4% (H)	-5.0% (V) -5.3% (H)	-1.8% (V) -14.7% (H)	4.2% (V) -16.6% (H)	4.2% (V) -16.6% (H)
Flexural Strength	D790	-9.0% (V) -8.2% (H)	-6.8% (V) -3.6% (H)	-17.2% (V) -14.4% (H)	-21.6% (V) -16.8% (H)	-21.6% (V) -16.8% (H)
Compressive Strength	D695	-4.7% (V)* -8.6% (H)*	29.2% (V)* 9.7% (H)*	-0.8% (V) 15.4% (H)	15.7% (V) 8.1% (H)*	15.7% (V) 8.1% (H)*
Izod notched impact	D256	-9.8% (V) 21.7% (H)	7.2% (V) 32.1% (H)	9.7% (V) -4.6 (H)	19.0% (V) 24.3% (H)	19.0% (V) 24.3% (H)
Shore Hardness	D2240	1.0%	0.0%	1.2%	-0.7% *	-0.7% *

^†^ Testing was conducted by Westmoreland Mechanical Testing & Research, Inc., an A2la ISO 17,025 accredited and NADCAP accredited laboratory

* The exposed sample result is Not Statistically Significant compared to the unexposed control

Overall, the single simulated use exposure (Table 5) resulted in smaller percent changes than the worst-case chemical exposure (Table 4) for the test methods evaluated. For the isotropic Formlabs vat polymerization produced materials, minor differences were observed in horizontal versus vertical printing for compressive strength and Izod notched impact. For the anisotropic Stratasys material jetting produced materials, there were differences in tensile strength, compressive strength, and Izod notched impact based on print orientation. The results support the material compatibility of the 3D printed material for vaporized hydrogen peroxide sterilization, and the differences are not considered practically significant. Further, these results support the 15x chemical exposure as a worst-case compared to single, simulated use exposure.

**Table 5:** Mechanical property evaluations after simulated use exposure in the specialty cycle

Test Name	ASTM^†^	Material / % Change				
		Formlabs BioMed Amber	Formlabs BioMed Clear	Stratasys MED610	Stratasys MED615	Stratasys MED620
Tensile Strength	D638	-1.4*	-6.8	-7.5%	-9.8%	-9.8%
Compressive Strength	D695	-1.3%*	14.8%*	11.6%	2.1%	2.1%
Shore Hardness	D2240	0.7%	0.2%	0.7%	0.5%	0.5%

^†^ Testing was conducted by Westmoreland Mechanical Testing & Research, Inc., an A2la ISO 17,025 accredited and NADCAP accredited laboratory

* The exposed sample result is Not Statistically Significant compared to the unexposed control

### Dimensional analysis

For this study, the effect of a sterilization process on the dimensional accuracy of a 3D printed item has also been evaluated. 3DTAs manufactured with the Formlabs and Stratasys materials were evaluated before and after sterilization then analyzed for differences. After the post-processing analysis was complete, the data sets for the pre- and post-processing analyses were overlaid and compared for difference via a heat map generated by the PolyWorks Inspector Essentials software. Physical measurements of the test article were taken with calipers before and after processing. As shown in Table 6, vaporized hydrogen peroxide processed 3D printed devices are dimensionally stable as little to no changes were observed after sterilization.

**Table 6:** Dimensional analysis pre- and post-sterilization differences

Material	3D Scan Differences	Measured Differences
Formlabs BioMed Amber	≤ 0.5 mm	≤ 0.01 mm
Formlabs BioMed Clear	≤ 0.1 mm	≤ 0.01 mm
Stratasys MED610	≤ 0.2 mm	≤ 0.1 mm
Stratasys MED615	≤ 0.5 mm	≤ 0.1 mm
Stratasys MED620	≤ 0.5 mm	≤ 0.1 mm

### Temperature evaluation

Lastly, plastic devices were processed in the Specialty F Cycle (the longest Specialty Cycle) and had their temperatures taken immediately afterwards. A total of nine temperature measurements ranged from 48 to 53 °C immediately after sterilization. The maximum plastic device temperature had previously been 43 °C for similar cycles used to process reusable medical devices. Therefore, even with the slightly higher temperatures after the additional aeration of the longest Specialty Cycle, device temperatures maintained a temperature range considered ‘low temperature’ (below 60 °C) for medical device processing.

## Discussion

### Sterilization efficacy

Medical devices used in the surgical field must be sterilized to prevent nosocomial infections. Patient-specific devices that are 3D printed within a hospital have considerations beyond the devices that a hospital routinely sterilizes. For example, device contamination during and post-manufacture coupled with the potential of material voids formed within the device are novel concerns for 3D printed device sterilization. Importantly though, and unlike reusable medical devices, single-use 3D printed devices are not exposed to patient soils and clinically relevant, pathogenic organisms prior to sterilization.

While the vat polymerization and material jetting produced parts without voids, other printing methods can contain significant porosity that may be of concern for sterilization. Research by Popsecu et al. [9] studied the disinfection and decontamination of devices 3D printed with ABS filament via material extrusion which creates parts layer by layer. Despite build optimization and solvent treatment with acetone vapor post-production, significant porosity remained on the devices and allowed liquid infiltration.

The potential for viable microorganisms within 3D printed device voids is a concern as the printing process can theoretically seal microorganisms within the void. Should a device be damaged during use, as shown by Shea et al., [10] there is a risk for patient exposure to an entrained microorganism. This raises questions about the potential bioburden on a 3D manufactured device as well as the post-processing sterility of those devices. Wangsgard and Winters reported that for 3D printed devices, bioburden levels would be low due to heat in the 3D print process [11]. Neches et al. identified that parts printed via material extrusion were sterile post-processing when taken from the printer and immersed within a growth media [12]. This effect was attributed to the temperature of the process, which, at 190–240 °C, is hotter than many decontamination and sterilization processes. When contamination was observed, it was determined to have been caused by post-print handling as the contaminants, *Staphylococcus epidermidis* and *Propionibacterium acnes*, are common to human skin. Lastly, in a study by Aguado-Maestro et al., five material extrusion printed cylinders were directly inoculated during a halt in the printing process with > 10^8^ CFU *Staphylococcus epidermidis* [13]. The print process was resumed sealing the cylinders, and the cylinders were incubated in growth media to evaluate for organism growth. Four of the five cylinders showed growth of 2–12 CFU of organism while one cylinder had no growth; a greater than 7-log reduction.

To limit risk of ineffective sterilization, 3D medical device manufacturers need to (1) select materials/print methods that minimize the presence of voids and (2) develop a system to control the microbial quality of the device prior to sterilization.

### Biocompatibility

3D printed medical devices must be demonstrated to be biocompatible to protect patients from biological risks from the device. Medical devices have been 3D printed for many years [[14]. More recently it is hospitals that are manufacturing the 3D printed medical devices [4, 15, 16]. It is the device manufacturer’s responsibility to establish biocompatibility of the finished device. There are a multitude of factors than can affect biocompatibility of a 3D printed medical device including the material, curing, cleaning, support removal, and sterilization process.

The importance of following printer manufacturer printing and processing guidelines cannot be minimized. The material selection is straightforward as printer manufacturers identify specific materials qualified as biocompatible for different applications. The instructions for use of solvents for residual resin removal, support removal, and cleaning is validated by the manufacturer. All processing instructions must be strictly followed to ensure biocompatibility. One risk to not following manufacturer’s instructions is the risk of insufficiently cured or polymerized acrylates (the resin used to create the part) as acrylates are a recognized health concern [17–19].

The impact of sterilization on biocompatibility has not been as well characterized as other process steps. With steam sterilization identified as the final processing step, there may be an assumption that sufficient biocompatibility data exists to support that the sterilization process had no effect on the biocompatibility of the processed devices; hence, biocompatibility testing may not have been conducted. While a change in physical properties or appearance after steam sterilization can be identified in a visual inspection process, a change in biocompatibility cannot, so an understanding of the device biocompatibility after sterilization should be considered.

### Material evaluations

Steam sterilization (at 121 °C, 132 °C, or 134 °C) is the most common method used to sterilize 3D printed parts in a hospital today [20–22]. The impact of steam sterilization on a variety of printed device materials has been evaluated by many researchers with widely varying results. For example, while Shaheen et al. and Marei et al. found steam sterilization to be less reliable and cause physical changes to the device, Torok et al. did not identify any difference after steam sterilization [20–22]. The benefit of vaporized hydrogen peroxide sterilization is that it is a low temperature method with a maximum temperature of less than 60 °C. While a lower process temperature is generally less damaging to materials, there is limited data for comparing the VH2O2 sterilization results presented in this report to other sterilization modalities.

One study that can be compared to data from this evaluation was published by Van Dal, who evaluated Formlabs BioMed Clear tensile bars printed at angles of 0° and 45° [23]. After cleaning with an automated washer, the materials were steam sterilized at 134 °C for 3.5 min. The percent change pre- and post-sterilization is shown in Table 7 where % change is calculated in same manner as for Tables 4 and 5: [(exposed-unexposed)/unexposed x 100%]. The steam processed tensile strength bars printed at 0° were warped after processing and showed some delamination; both defects were attributed to peel forces from the 0° print orientation. Note that no materials defects were observed for the materials evaluated in this report, inclusive of the BioMed Clear tensile bars processed in the Specialty Cycle.

The Van Dal results in Table 7 are best compared to single sterilization cycle (simulated use) results in Table 5 for BioMed Clear for tensile strength where the tensile strength was very similar after vaporized hydrogen peroxide and steam. For the flexural and impact strength evaluations, comparing 0° print (Table 7) to the horizontally printed (H) BioMed Clear results (Table 4), shows that flexural strength did not significantly change after vaporized hydrogen peroxide but increased after steam while impact strength increased after vaporized hydrogen peroxide but decreased after steam.

**Table 7:** Van Dal evaluation of formlabs BioMed clear

Test Name	Steam Sterilization 134 °C, 3.5 min		This study
	0° Print, % change	45° Print, % change	
Tensile Strength	-5.8	1.4	-5.3% (H)
Flexural Strength	31	30	-3.6% (H)
Notched Impact Strength	-17	-21	32.1% (H)

A separate study by Torok et al., evaluated Stratasys MED610 in a surgical guide configuration for Tensile Strength, Flexural Strength, and Hardness after steam sterilization at 121 °C (20 min) and 134 °C (10 min) [22]. The % change results are shown in Table 8 with % change calculated from report data in same manner as for Tables 4 and 5. The authors further determined that there was no significant difference pre- and post-steam sterilization via scanning electron and stereomicroscopic examinations. Steam sterilization at 134 °C for 10 min was noted to cause deformations while no materials defects were observed for the materials evaluated in this report, inclusive of the MED610 ASTM coupons processed in the Specialty Cycle.

The Torok et al. 121 °C steam data is most directly compared to MED610 data in Table 5 where tensile strength after sterilization was similar. Comparing 121 °C steam data (Table 8) to the MED610 results (Table 4), flexural strength did not significantly change after steam but decreased after vaporized hydrogen peroxide while hardness changed minimally after steam or vaporized hydrogen peroxide sterilization.

**Table 8:** Torok et al. evaluation of stratasys MED610 in steam

Test Name	Sterilization Method / % Change		This study
	121 °C Steam. 20 min % change	134 °C Steam, 10 min % change	
Tensile Strength	-4%	15%	-1.8% (V) -14.7% (H)
Flexural Strength	0 to -7%	-44 to 14	-17.2% (V) -14.4% (H)
Hardness	-2%	12%	1.2%

The ability to terminally sterilize 3D printed devices at lower processing temperatures than steam, e.g., with vaporized hydrogen peroxide, may allow for new materials selection, in particular for materials than cannot withstand the high temperatures of steam sterilization.

### Dimensional analysis

Understanding the impact a sterilization process has on device dimensions is critical as a 3D printed anatomical model may be used diagnostically and a surgical guide will be used intraoperatively. The critical considerations and contributions to consider when measuring and evaluating the accuracy of 3D printed medical models has been detailed by George et al. [24].

The contribution of sterilization to the workflow process, although not directly considered by George et al., is especially important with plastics when considering a low temperature sterilization method like vaporized hydrogen peroxide (temperatures 60 °C or lower) in contrast to steam sterilization (temperatures of 121–134 °C). Many studies have evaluated the effect of steam sterilization on the dimensionality of 3D printed items with mixed results dependent on material and exposure temperatures as discussed within the physical properties results (Torok, Marei, and Shaheen).

This study demonstrated that low temperature vaporized hydrogen peroxide processed 3D printed devices are dimensionally stable; little to no changes were observed after sterilization: ≤ 0.5 mm for pre- and post-processing scans overlaid and compared for difference via a heat map. Similarly, other researchers have identified only minimal dimensional changes on devices after vaporized hydrogen peroxide. Toro et al. evaluated the dimensionality of material extrusion printed ABS anatomical models and guides before and after evaluation with vaporized hydrogen peroxide [25]. Their dimensional analysis found that post-sterilization the mean differences between the printed pieces and original design were within the 95% confidence interval of -0.096 to -0.094 mm for models and 0.140 to 0.141 mm for guides; thus maintaining dimensional stability after sterilization. The biological evaluation showed that after sterilization these devices were not cytotoxic, pyrogenic, or sensitizers, and had no acute systemic toxicity.

### Regulatory implications

A device manufacturer is responsible for ensuring that each device meets its intended design requirements (form, fit, and function). This responsibility spans the entire life cycle of the medical device; that is, from manufacture, including post-production processing (e.g., cleaning, curing and sterilization), through use in a patient procedure.

Hospital-based3D printed medical device is relatively new when compared to medical devices 3D printed by companies and sold to hospitals. Authors have reviewed the concept of 3D printing within the context of current regulations [5, 26–29]. In December 2021, the US FDA published a discussion paper for comment as they seek to create a regulatory framework for this new application [30]. As a practical matter, organizations such as Radiological Society of North America (RSNA) have worked collaboratively to identity and define what the 3D device quality system process in a hospital would entail [4, 15, 31]. Regardless of the eventual regulatory and quality system particulars, there will be an obligation upon the 3D printed medical device manufacturer to establish within that process a sterilization method that ensures sterilization efficacy, biocompatibility, and material compatibility. Within the context of a quality system, data supporting sterilization may prove useful, as would the methodologies used to support FDA clearance of this particular workflow.

## Conclusion

A vaporized hydrogen peroxide sterilization method with the Specialty Cycle was used to evaluate sterilization, biocompatibility and material compatibility of select Formlabs and Stratasys materials. The test results, and a comparison to other relevant sterilization methodology results, support the use of low temperature vaporized hydrogen peroxide to sterilize 3D printed surgical guides and anatomical models. The test result data was used in support of regulatory validation and clearance [1].

### Trademarks

V-PRO™ and VAPROX™ are trademarks of STERIS, its affiliates or related companies.

All other product and company names referenced are trademarks of their respective owners.
